# Management of the Bleeding Patient Receiving New Oral Anticoagulants: A Role for Prothrombin Complex Concentrates

**DOI:** 10.1155/2014/583794

**Published:** 2014-07-20

**Authors:** Lisa M. Baumann Kreuziger, Joseph C. Keenan, Colleen T. Morton, David J. Dries

**Affiliations:** ^1^University of Minnesota, 420 Delaware Street SE, Minneapolis, MN 55455, USA; ^2^Regions Hospital, University of Minnesota, 640 Jackson Street, No. 11503C, Saint Paul, MN 55101, USA

## Abstract

Ease of dosing and simplicity of monitoring make new oral anticoagulants an attractive therapy in a growing range of clinical conditions. However, newer oral anticoagulants interact with the coagulation cascade in different ways than traditional warfarin therapy. Replacement of clotting factors will not reverse the effects of dabigatran, rivaroxaban, or apixaban. Currently, antidotes for these drugs are not widely available. Fortunately, withholding the anticoagulant and dialysis are freqnently effective treatments, particularly with rivaroxaban and dabigatran. Emergent bleeding, however, requires utilization of Prothrombin Complex Concentrates (PCCs). PCCs, in addition to recombinant factor VIIa, are used to activate the clotting system to reverse the effects of the new oral anticoagulants. In cases of refractory or emergent bleeding, the recommended factor concentrate in our protocols differs between the new oral anticoagulants. In patients taking dabigatran, we administer an activated PCC (aPCC) [FELBA] due to reported benefit in human in vitro studies. Based on human clinical trial evidence, the 4-factor PCC (Kcentra) is suggested for patients with refractory rivaroxaban- or apixaban-associated hemorrhage. If bleeding continues, recombinant factor VIIa may be employed. With all of these new procoagulant agents, the risk of thrombosis associated with administration of factor concentrates must be weighed against the relative risk of hemorrhage.

## 1. Introduction

Hemorrhage is the major cause of early mortality after injury and a leading risk in any operative procedure. Recently developed target-specific oral anticoagulants defy traditional reversal protocols previously used with warfarin. In the face of new oral anticoagulants, we have developed additional approaches to management of bleeding. Patients with life threatening hemorrhage may benefit from use of a new 4-factor prothrombin complex concentrate (PCC) (Kcentra, CSL Behring Gmbh, Marburg, Germany). Recent data suggest that there may be a role for factor concentrates including PCCs, activated PCCs (aPCC), and recombinant factor VIIa (rfVIIa, NovoSeven, Novo Nordisk, Bagsvaerd, Denmark) in dabigatran, rivaroxaban, and apixaban-associated bleeding. This report will briefly review the mechanism of action of the oral anticoagulants, present our bleeding management protocols, and discuss the rationale for our use of prothrombin complex concentrates and rfVIIa in refractory hemorrhage.

## 2. Mechanism of Action

Understanding the mechanism of action of oral anticoagulants provides a biologic foundation for use of PCCs, aPCCs, and rfVIIa in treatment of uncontrolled bleeding. Warfarin creates a deficiency of factors II, VII, IX, and X through inhibition of vitamin K-dependent carboxylation of the clotting proteins [1]. Replacement of functional coagulation factors can reverse the anticoagulant effect of warfarin. The newer oral anticoagulants interact with the coagulation cascade in different ways. Dabigatran directly inhibits thrombin (activated factor II), which limits the formation of fibrin [2]. Rivaroxaban and apixaban directly inhibit activated factor X [3, 4]. Replacement of clotting factors will not reverse the effects of dabigatran, rivaroxaban, and apixaban and we currently do not have antidotes for these drugs.

Rates of bleeding seen with the new oral anticoagulants in real world patients treated with dabigatran are consistent with those seen in clinical trials and less than those seen with warfarin. Most of the data available at this time comes from clinical trials. Gastrointestinal and intracranial hemorrhage are the two most important bleeding complications reported with the new oral anticoagulants. In postmarketing surveillance, there did not appear to be higher bleeding rates associated with dabigatran as opposed to warfarin. Some of the largest trials are in patients with atrial fibrillation. In these studies, reduced rates of intracranial hemorrhage are seen with dabigatran in comparison to warfarin as well as a reduced death rate. The rate of gastrointestinal hemorrhage is similar between dabigatran and warfarin-treated patients. Factor Xa inhibitors are also associated with reduced risk of mortality and intracranial hemorrhage compared to warfarin in trials conducted in patients with atrial fibrillation and may be used in ablation procedures [5–8].

PCCs are plasma-derived products containing factors II, VII, IX, and X. The 3-factor PCCs contain a minimal amount of factor VII.  Table 1 shows the PCCs available in the United States. FEIBA (Baxter AG, Vienna, Austria) is unique because it contains factor VII primarily in the activated form (activated PCC (aPCC)) [9]. Kcentra contains all of the vitamin K-dependent proteins (factors II, VII, IX, and X and proteins C and S) [10]. Kcentra and Bebulin (Baxter AG, Vienna, Austria) contain small amounts of heparin which is insufficient to cause anticoagulation but contraindicates use of these products in patients with a history of heparin-induced thrombocytopenia. Recombinant factor VIIa initiates coagulation independent of tissue factor, factor VIII and factor IX, and is approved for use in patients with factor VII deficiency and hemophilia with factor inhibitors [11]. PCCs provide replacement of functional vitamin K-dependent proteins to more rapidly reverse the anticoagulant effect of warfarin [12]. In contrast, for patients with uncontrolled bleeding while taking dabigatran, rivaroxaban, or apixaban, PCCs, aPCCs, and rVIIa can be used to activate the coagulation system.

## 3. Animal and Human Data for Management of Dabigatran, Rivaroxaban, and Apixaban-Associated Hemorrhage

The PCC selection in our protocols for dabigatran, rivaroxaban, and apixaban-associated hemorrhage is based upon the available human and animal data presented in detail in Table 2 and summarized in Table 3. Human in vitro and animal studies have shown improved thrombin generation after administration of aPCCs in dabigatran treated animals [13, 14]. In a prospective crossover study, 4-factor PCCs failed to correct coagulation times or thrombin generation in humans taking dabigatran [15] suggesting that 4-factor PCCs are not useful for dabigatran-associated hemorrhage. rfVIIa could be considered in dabigatran-associated bleeding based on corrected time to thrombin generation from in vitro studies and decreased bleeding time in rats [13, 14, 16]. However, one case report suggested decreased bleeding after administration of rfVIIa in a patient taking dabigatran [17] and two reports noted continued bleeding [18–20]. Due to the mixed efficacy reported in case reports of rfVIIa, we use the aPCC (Feiba) prior to rfVIIa in refractory bleeding associated with dabigatran [21, 22]. In contrast to dabigatran, 4-factor PCCs improved rivaroxaban-induced coagulation testing abnormalities in a prospective crossover study in healthy volunteers [15], informing our decision to use a 4-factor PCC (Kcentra) in the setting of rivaroxaban-associated refractory bleeding. Given the mechanistic similarity and positive in vitro data, we also use a 4-factor PCC (Kcentra) in patients with refractory apixaban-associated bleeding [23]. Animal studies suggest that correction of coagulation testing does not always correlate to improvement in bleeding outcomes [14]. Outcomes of patients receiving factor concentrates via these protocols must be closely monitored.

## 4. Protocols

**Figure 1:**
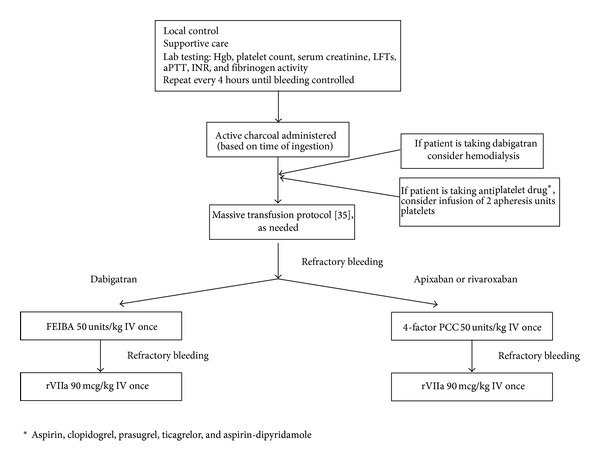
Management protocol for hemorrhage in patients taking dabigatran, rivaroxaban, or apixaban.

Our institution has created protocols to help direct the use of factor concentrates in the treatment of life threatening bleeding in patients taking new oral anticoagulants (Figure 1). The initial measures are the same for any bleeding patient, with local intervention and supportive care. In addition, confirmation of medication, dosing, and duration since the last dose guides further therapy. Renal and hepatic function are evaluated to determine patient metabolism of medication. Transfusion of packed RBCs and a transfusion protocol featuring a balance of packed red blood cells, plasma, and platelets may be utilized depending on the severity of hemorrhage. Based largely on retrospective data, the optimal ratio of plasma to packed RBCs administered is thought to be 1 : 1 or 1 : 2 [24]. The effects of antiplatelet agents are reversed by transfusion of 2 apheresis units of platelet concentrates if needed (see below) [25, 26].

Our management protocols in the setting of life threatening bleeding in the patient taking dabigatran, rivaroxaban, or apixaban are summarized in Figure 1. Activated charcoal may be administered if the last dose of anticoagulant was ingested less than 2 hours (dabigatran [2] or rivaroxaban [27]) or 3 hours (apixaban [28]) before presentation. Hemodialysis removes dabigatran and should be considered based on clinical status and need for surgical intervention. In a survey of nonmalignant hematologists, withholding the anticoagulant and dialysis were the most effective treatment used in 80% of bleeding episodes associated with rivaroxaban and dabigatran, respectively [29]. Thus, the use of specialized resuscitation protocols is often not required.

After transfusion of 4 units of PRBCs, we transfuse PRBCs, plasma, and platelets in a 1 : 1 : 1 ratio with a goal of hemoglobin of 9-10 g/dL [24]. We employ this transfusion strategy to avoid additional mechanisms of coagulopathy as the new oral anticoagulants prolong the PTT and INR and, thus, interfere with laboratory monitoring of other coagulation abnormalities. For patients on antiplatelet drugs we consider administration of two apheresis units of platelets.

In cases of refractory bleeding, the recommended factor concentrate in our protocols differs between the new oral anticoagulants. In patients taking dabigatran, we administer 50 units/kg IV of aPCC (Feiba) due to suggested benefit in human in vitro studies [13]. Based on human clinical trial evidence, 50 units/kg IV of the 4-factor PCC (Kcentra) is suggested for patients with refractory rivaroxaban or apixaban-associated hemorrhage. If bleeding continues, rfVIIa may be employed. Thrombosis has been reported with administration of all of the factor concentrates and the relative risk of hemorrhage and thrombosis must be considered.

Platelets are an essential but poorly understood component of hemostasis after injury. Previous evidence identifies admission platelet counts as inversely correlated with early mortality and supports transfusion of platelets with critical injury and trauma, even for platelet counts in the normal reference range. Quantitative platelet deficits have predicted progression of intracranial hemorrhage and mortality after traumatic brain injury. Study of platelet dysfunction has been hindered by technical complexity of existing platelet assays [30, 31].

Given the controversy which continues in the trauma and acute surgery literature, a number of observations can be made [30, 32–37]. Patients receiving antiplatelet agents have an increased number of comorbidities. While recent retrospective work suggests limited impact of antiplatelet medications on outcome with trauma, limited prospective work suggests that the elderly and patients with intracranial hemorrhage are at greater risk for poor outcomes if taking antiplatelet agents. A normal platelet count is not reassuring in this setting. Normal clotting studies also do not predict good outcome. Despite this, a specific role for empiric platelet therapy has not been identified. Finally, while a variety of assays for platelet function have been reported, consensus regarding the optimal assay and the standard for acute management of injury has not been reached. Of assays which can be performed at the bedside, thromboelastography is the most promising bedside assay [31]. Our recommendation favoring consideration of platelet administration in the setting of life threatening bleeding must be understood in the context of the quality of studies which are available.

## 5. Conclusion

Our institution developed management protocols in an effort to standardize treatment of severe bleeding associated with use of new oral anticoagulants and approval of new factor concentrates. These protocols are based on limited human data but serve as a tool to guide therapy as providers gain experience with these anticoagulants and PCCs. Standardizing therapy allows the collection of clinical data, which can guide further trials. We anticipate continued modification of these protocols as laboratory and clinical experience expand. In particular, thrombosis risk with powerful newer agents designed to enhance coagulation must be monitored. At this time, data related to thrombosis risk is limited to anecdotes [5].

## Figures and Tables

**Table 1:** Prothrombin complex concentrates (PCC) available in the United States.

Product	Coagulation factors in product	FDA approved indications to manage hemorrhage	Heparin in product?
Nonactivated			
3-factor PCC			
Profilnine	Nonactivated II, IX, X, and small amounts of VII	Hemophilia B	No
Bebulin	Nonactivated II, IX, X, and small amounts of VII	Hemophilia B	Yes
4-factor PCC			
Kcentra	Nonactivated II, VII, IX, X, protein C, and protein S	Warfarin	Yes
Activated PCC			
Feiba	Activated VII, nonactivated II, IX, and X	Hemophilia A & B with inhibitors	No

**Table 2:** Animal and human studies using factor concentrates to reverse anticoagulant effect of dabigatran, rivaroxaban, and apixaban.

Author	Reversal agent	Test system	Coagulation test effect	Bleeding outcome effect
Dabigatran				
Van Ryn et al. [16]	aPCC	Rat	aPTT unchanged	Decreased bleeding time
	rfVIIa	Rat	aPTT decreased	Decreased bleeding time
Van Ryn et al. [14]	4-Factor PCC	Rat	PT decreased, aPTT unchanged	Decreased bleeding time
	aPCC	Rat	PT decreased, aPTT unchanged, increased thrombin generation	Decreased bleeding time
	rfVIIa		PT and aPTT decreased	Decreased bleeding time
Lambourne et al. [38]	4-Factor PCC	Mouse	No effect	No effect
	rfVIIa	Mouse	aPTT decreased	No effect
	4-Factor PCC + rfVIIa	Mouse	TT and aPTT decreased	No effect
	aPCC	Mouse	No effect	No effect
Pragst et al. [39]	4-Factor PCC	Rabbit	PT decreased, aPTT unchanged	Normalized blood loss
Zhou et al. [40]	4-Factor PCC	Mouse ICH		Prevented hematoma expansion; control level mortality
	rfVIIa	Mouse ICH		Ineffective control of hematoma expansion
Marlu et al. [13]	4-Factor PCC	Human in vitro	Increased amount of thrombin generation	
	rfVIIa	Human in vitro	Corrected time to thrombin generation	
	aPCC	Human in vitro	Corrected time to thrombin generation	
Eerenberg et al. [15]	4-Factor PCC	Human	No effect on aPTT, TT, or ECT	
Khoo et al. [41]	aPCC	Human in vitro	Increased thrombin generation and corrected time to thrombin generation	
Warkentin et al. [17]	rfVIIa	Human case report	Decreased aPTT and PT	Decreased blood loss
Garber et al. [18]	rfVIIa	Human case report		Worsening ICH
Truumees et al. [19]	rfVIIa	Human case report		Continued blood loss
Lillo-Le Louët et al. [20]	4-Factor PCC + rfVIIa	Human case report	PT and aPTT unchanged	Continued bleeding
	4-Factor PCC + rfVIIa	Human case report	PT and aPTT unchanged	Bleeding stopped with dialysis
Wychowski and Kouides [42]	3-Factor PCC	Human case report	TT and aPTT unchanged, PT decreased	No further bleeding
Dumkow et al. [43]	3-Factor PCC	Human case report		Clinical bleeding and hemoglobin stabilized
Rivaroxaban				
Perzborn and Tinel [44]	4-factor PCC	Rat	PT decreased	Bleeding time decreased
Godier et al. [45]	4-factor PCC	Rabbit	aPTT normalized, PT decreased	No effect on blood loss
	rfVIIa	Rabbit	aPTT normalized, PT decreased	Decreased bleeding time, no effect on blood loss
Perzborn et al. [46]	4-Factor PCC	Rat	Decreased PT, normalized TAT concentration	Reduced bleeding time
	aPCC	Rat	Decreased PT	Reduced bleeding time
		Primate	Reduced PT	Normalized bleeding time
	rVIIa	Rat	Decreased PT	Reduced bleeding time
		Primate	Decreased PT	Bleeding time unchanged
Marlu et al. [13]	4-Factor PCC	Human in vitro	Increased amount of thrombin generation	
	rfVIIa	Human in vitro	Corrected time to thrombin generation	
	aPCC	Human in vitro	Corrected all thrombin generation parameters	
Eerenberg et al. [15]	4-Factor PCC	Human in vivo	Normalized PT and thrombin generation	
Dinkelaar et al. [47]	4-Factor PCC	Human in vitro	No effect on PT or time to thrombin generation Normalized amount of thrombin generation	
Körber et al. [48]	4-Factor PCC	Human in vitro	No effect on aPTT, PT	
	rfVIIa	Human in vitro	Decreased clotting time, no effect on aPTT, PT	
Apixaban				
Escolar et al. [23]	PCC	Human in vitro	Increased thrombin generation	
	rfVIIa	Human in vitro	Increased thrombin generation	
	aPCC	Human in vitro	Increased thrombin generation	

aPCC: activated prothrombin complex concentrate; rfVIIa: recombinant factor VIIa.

**Table 3:** Summary of animal and human data for reversal of dabigatran, rivaroxaban, and apixaban using factor concentrates.

	Dabigatran		Rivaroxaban		Apixaban	
	Animal	Human	Animal	Human	Animal	Human
3-factor PCC		Case report +/−				
4-factor PCC	Rats +/− Rabbits + Mice − Mice ICH +	In vitro + In vivo − Case report −	Rats + Rabbits +/−	In vitro +/− In vivo +		In vitro +
aPCC	Rats +/− Mice −	In vitro +	Rat + Primate +	In vitro +		In vitro +
rfVIIa	Rats +/− Mice +/− Mice ICH −	In vitro + Case report +/−	Rat + Rabbits +/− Primate +/−	In vitro +/−		In vitro +

+: effective; −: ineffective; +/−: mixed results between studies or between coagulation testing and bleeding outcomes; PCC: prothrombin complex concentrate; aPCC: activated prothrombin complex concentrate; rfVIIa: recombinant factor VIIa.
